# Time-Frequency Based Phase-Amplitude Coupling Measure For Neuronal Oscillations

**DOI:** 10.1038/s41598-019-48870-2

**Published:** 2019-08-27

**Authors:** Tamanna T. K. Munia, Selin Aviyente

**Affiliations:** Michigan State University, Department of Electrical and Computer Engineering, East Lansing, MI- 48824 USA

**Keywords:** Dynamical systems, Biomedical engineering

## Abstract

Oscillatory activity in the brain has been associated with a wide variety of cognitive processes including decision making, feedback processing, and working memory. The high temporal resolution provided by electroencephalography (EEG) enables the study of variation of oscillatory power and coupling across time. Various forms of neural synchrony across frequency bands have been suggested as the mechanism underlying neural binding. Recently, a considerable amount of work has focused on phase-amplitude coupling (PAC)– a form of cross-frequency coupling where the amplitude of a high frequency signal is modulated by the phase of low frequency oscillations. The existing methods for assessing PAC have some limitations including limited frequency resolution and sensitivity to noise, data length and sampling rate due to the inherent dependence on bandpass filtering. In this paper, we propose a new time-frequency based PAC (t-f PAC) measure that can address these issues. The proposed method relies on a complex time-frequency distribution, known as the Reduced Interference Distribution (RID)-Rihaczek distribution, to estimate both the phase and the envelope of low and high frequency oscillations, respectively. As such, it does not rely on bandpass filtering and possesses some of the desirable properties of time-frequency distributions such as high frequency resolution. The proposed technique is first evaluated for simulated data and then applied to an EEG speeded reaction task dataset. The results illustrate that the proposed time-frequency based PAC is more robust to varying signal parameters and provides a more accurate measure of coupling strength.

## Introduction

The human brain has been modeled as a complex network with distributed topology. This distributed topology results in parallel and specialized information processing. Therefore, there is a need for a neural mechanism that enables information integration across specialized brain regions. Various forms of neural synchrony between oscillations across different frequency bands have been suggested as the major mechanism of neural integration. Previous studies based on electrophysiological measurement of neural activity suggest that different frequency bands are responsible for distinct computational roles^1^ as oscillations are thought to create synchronization across specialized brain regions to corroborate cognitive processing^2,3^. The power and/or the synchronization measured across different frequency bands have been related to various cognitive and neuronal functions^4–6^. For example, the gamma band neuronal activity in the human brain has been demonstrated to play an important role in visual perception^7,8^, whereas alpha band oscillations in the occipital region were interpreted to be an indicator of reduced visual attention^1^. Most recently, emerging evidence from various studies suggests that oscillations from different frequency bands are not isolated and independent; consequently, they can interact with each other in the form of modulation^9,10^. This interaction of oscillations across different frequency bands is referred to as “cross-frequency coupling”^11–14^. Multiple forms of cross-frequency coupling have been analyzed including phase/amplitude^12,15–17^; phase/phase^18–21^; amplitude-to-amplitude^22,23^; and phase-frequency^11,14^. Phase-amplitude coupling (PAC) is the most-studied type of cross-frequency coupling and is thought to be responsible for integration across populations of neurons^24,25^. Low frequency brain activity controls the information exchange between brain regions by modulating the amplitude of the high frequency oscillations. PAC thus quantifies the modulation of the amplitude of high frequency oscillation, typically 30–100 Hz, with the phase of slow rhythm, typically 5–12 Hz^26,27^.

Phase-amplitude coupling has been detected in different species including EEG, MEG and ECoG recordings of the human brain^15,22,24,28^, LFP of mice^29,30^, rats^26^, sheep^31^, monkeys^32^ and within various brain regions such as the hippocampus^28–30,32^, the neocortex^10,32,33^, and basal ganglia^15^. The neural information processing and cognitive functioning, particularly sensory signal detection^34^, attention^16^, visual perception^35,36^ and memory processing^17,28^ have also been shown to exhibit dynamic PAC. More recently, disruption in PAC patterns has been linked to various neurological disorders, such as, autism spectrum disorder^37^, schizophrenia^38^, and Parkinson’s Disease^39^.

Conventional methods for computing PAC rely on the Hilbert transform of the bandpass filtered neurophysiological data for extracting the analytic signal with the instantaneous amplitude and phase components. The conventional approach to PAC computation is composed of the following three steps^27,40,41^: (1) bandpass filtering the input data into bands of interest, e.g., theta and gamma; (2) applying Hilbert transform to extract amplitude and phase time series from each frequency band of interest; and (3) quantifying the relationship between the phase and the amplitude time series. However, Hilbert transform based amplitude and phase estimations suffer from some shortcomings which can influence the final PAC estimates and the subsequent neuroscientific findings^40,42,43^. Most of these limitations arise from the first step of the processing pipeline, i.e. bandpass filtering. It is well-known that the use of Hilbert transform based amplitude and phase estimation relies on the assumption that the signals are narrowband, i.e., nearly sinusoidal^40,44^. The narrowband assumption is questionable for high frequency oscillations. Therefore, using the Hilbert transform for extracting amplitude time series may result in non-meaningful amplitude estimates for PAC computation^45–47^. To extract narrowband signals from neural oscillations, Hilbert transform based methods rely on bandpass filtering the signals before estimating amplitude and phase. Moreover, it is advisable to use a wideband filter for high frequency oscillations and a narrowband filter for low frequency oscillations^48^. Therefore, in conventional PAC computation, a systematic bias arises due to the selection of the various bandpass filter parameters, including the bandwidth, order of the filter, and transition band. Prior research indicates that the bandwidth for the high frequency component should be at least twice as high as the low frequency component in order to capture the amplitude modulation effect^45,48^. Therefore, the bandwidth is proportional to the center frequency, and this choice can lead to a systematic bias. Moreover, Hyafil *et al*.^46^ also showed that certain bandwidth choices might mistakenly compute phase-frequency coupling as PAC or produce erroneous amplitude-amplitude coupling. More recently, generalized Morse wavelet (GMW) has been proposed as an alternative to Hilbert transform for extracting the amplitude and phase components^49,50^ to address these limitations. GMWs are analytic wavelets, thus convolving the wavelet with the signal converts the signal to its analytic representation and is equivalent to bandpass filtering with a filter whose bandwidth scales with frequency. In this manner, the wavelet transform based PAC measure avoids the problem of designing the best filter as the filter bandwidth is automatically controlled by the wavelet scale. Estimating PAC from GMWs proceeds in the same way after the instantaneous amplitude and phase are extracted from the wavelet transform. Even though the wavelet transform addresses the problem of bandpass filtering, it suffers from a number of other problems including the choice of different design parameters in GMW^50^. Moreover, the lower and upper frequency and the scale parameters need to be chosen carefully to make sure that the peaks of the wavelet’s frequency spectrum occur at the frequency of interest. Therefore, if the user does not choose the input frequency range or the number of scales appropriately, the ridges may not be as apparent. Consequently, PAC estimation may suffer from this.

For the last step in the pipeline for quantifying PAC, various indices quantifying the modulation between the phase and amplitude time series have been proposed. This relationship has been studied by mean vector length (MVL) which quantifies the circular variances through the magnitude of the mean of the complex composite signal^10^, modulation index (MI) which quantifies the deviation of the phase-amplitude distribution from the uniform distribution through Kullback-Leibler divergence^27^, and phase locking value (PLV) which computes the circular variance of the consistency of the phase differences between the phase of the low frequency signal and the phase of the amplitude of the high frequency signal^51^. Extensive evaluation of these metrics shows that while MI is robust against noise and short data epochs, MVL performs better at high SNR^41^.

In this article, we present a novel method for assessing PAC based on a high resolution complex time-frequency distribution. First, we introduce an instantaneous phase and amplitude estimation method based on RID-Rihaczek distribution^52,53^. The properties of this quadratic time-frequency distribution, such as time marginal and energy preservation, are used to estimate both the envelope of the high frequency oscillation and the phase of the low frequency oscillation. This approach replaces the Hilbert transform and the analytical wavelet transform for extracting the amplitude and phase. Unlike Hilbert transform based methods, the proposed method obtains the analytic signal without any bandpass filtering. Unlike the wavelet transform, this approach results in uniformly high resolution across time and frequency and does not depend on the choice of different input parameters. We then compute MVL based on the extracted amplitude and phase time series to quantify PAC. It is important to note that even though the current paper focuses on MVL to quantify the PAC, it is possible to combine the amplitude and phase estimates obtained from RID-Rihaczek distribution with other metrics such as MI or PLV. The proposed method is first tested on simulated data and evaluated in terms of its resolution, accuracy of estimating the coupling strength and robustness against varying signal parameters. Finally, the method is applied to multi-channel EEG data recorded during a cognitive control study to determine differences between response types and to identify brain regions and frequency bands that show increased PAC.

## Materials and Methods

### Experimental data

To investigate the validity of the proposed PAC approach, experiments were first conducted on two sets of synthesized data, and then on a human EEG dataset collected from a cognitive control study published earlier^54^.

#### Synthesized dataset 1

In the first simulation, the sum of two sinusoids with known coupling parameters was generated to assess the accuracy of the proposed method. These two sinusoids referred to as phase and amplitude signals, are generated following Tort *et al*. as follows^27,55^:

1 $${\rm{Phase}}\,{\rm{signal}}:{x}_{{f}_{p}}(t)={K}_{{f}_{p}}\,\sin (2\pi {f}_{p}t),$$

2 $${\rm{Amplitude}}\,{\rm{signal}}:{x}_{{f}_{a}}(t)={A}_{{f}_{a}}(t)\sin (2\pi {f}_{a}t),$$

where, ${A}_{{f}_{a}}(t)={K}_{{f}_{a}}\frac{(1-\chi )\sin (2\pi {f}_{p}t-{\varphi }_{c})+\chi +1}{2}$, *χ* ∈ [0, 1], ${K}_{{f}_{p}}$ and ${K}_{{f}_{a}}$ are constants that determine the amplitude of the phase frequency (*f*_*p*_) and amplitude frequency (*f*_*a*_), respectively. *ϕ*_*c*_ is the phase of the low frequency phase providing signal at the time point where the magnitude of the amplitude signal bursts is maximum. *χ* is the fraction of amplitude signal that is not modulated by the phase signal, thus the coupling strength is given by (1 − *χ*). To generate the synthesized dataset 1, these two components were added:

3 $$x(t)={x}_{{f}_{a}}(t)+{x}_{{f}_{p}}(t)+\varepsilon (t),$$

where *ε*(*t*) is additive noise and was generated with a combination of random samples created through power law and white Gaussian noise. The power law samples simulate the background brain activity, and the white Gaussian samples simulate measurement noise^55^. The strength of the white Gaussian noise parameters was set to half of the strength of the power law samples.

The synthesized dataset was generated with the low frequency signal at 5 Hz, high frequency signal in the range of 50–70 Hz, sampling frequency of 1000 Hz, SNR levels ranging from −5 to 10 dB, coupling intensity in the range of 0.1 to 1.0, and data length in the range of 0.4 to 6 seconds. Finally, to test the effect of coupled high frequency, a 10 second signal was generated with SNR = 3 dB, sampling frequency = 1000 Hz, coupling intensity = 0.9, low frequency = 5 Hz, and changing the coupled high frequency from 10 Hz to 70 Hz with an increment of 5 Hz.

#### Synthesized dataset 2

The second synthesized dataset was generated using the fact that the spectrum of EEG data follows the power law, i.e., higher the frequency, weaker the amplitude $(P(f) \sim (\frac{1}{{f}^{\beta }}))$. The strength of the amplitude decrease is defined by the parameter *β* with *β* = 0, 1, and 2 indicating white noise, pink noise, and Brownian (red) noise, respectively^41^. The spectrum of EEG data is reported to be related to Brownian noise^56,57^, thus, the synthesized data was generated from Brownian noise. First, 10 seconds of Brownian noise data was generated at a sampling frequency of 1000 Hz following the method developed by Zhivomirov^58^. Next, the signal was bandpass filtered at low phase providing frequency with a bandwidth of 2 Hz for generating the phase signal. The same simulated signal was then bandpass filtered at high amplitude providing frequency to create the amplitude signal. The low frequency phase signal bandwidth was set to 8–10 Hz, and the high frequency amplitude signal bandwidth was set to 50–70 Hz. The coupling between phase and amplitude signals was generated using the procedure described by Kramer and Eden^59^. The time locations of relative maxima and minima of the phase signal were detected. At each maxima, a DC shifted Hanning window with a duration of 42 ms, i.e., the amplitude of the Hanning window is shifted by 1, was multiplied with the amplitude time series. The monophasic coupling was generated by multiplying the Hanning window with the amplitude time series centered at the relative maxima of the phase time series. The intensity of the phase-amplitude coupling is controlled by multiplying the Hanning window with a constant *I*, where *I* = 1 indicates full coupling and *I* = 0 indicates no phase-amplitude coupling. An additional frequency modulated noise was generated by generating Brownian noise of the same length, bandpass filtering the noise signal at similar low and high frequency bands and adding them to the phase and amplitude signals, respectively.

Synthesized data were generated with low frequency signal at 10 Hz, high frequency signal in the range of 50–70 Hz, sampling frequency of 1000 Hz, SNR levels ranging from −5 dB to 17 dB, coupling intensity in the range of 0.1 to 1.0, and data length in the range of 0.4 to 6 seconds. To test the effect of coupled high frequency, a 10 second signal was generated with SNR = 6 dB, sampling frequency = 1000 Hz, coupling intensity = 0.9, low frequency = 10 Hz, and changing the coupled high frequency from 15 Hz to 70 Hz with an increment of 5 Hz. Finally for visualizing the broadband vs narrow-band coupling effect, 10 second signals (SNR = 6 dB, sampling frequency = 1000 Hz, coupling intensity = 0.85) were generated with the *f*_*p*_ frequency fixed at 5 Hz and varying the *f*_*a*_ frequency range as [65–75], [60–80], [55–85], [50–90], [45–95] and [40–100] to generate 10 Hz to 60 Hz coupling bandwidths.

**Figure 1:**
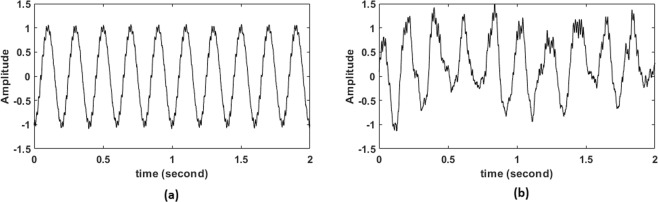
Representation of the synthesized data generated for the analysis; (**a**) Simulated signal generated through the method described in Synthesized Dataset 1 subsection; (**b**) Simulated signal generated through the method described in Synthesized Dataset 2 subsection. Both signals were generated for 2 seconds with a sampling frequency = 1000 *Hz*, *f*_*p*_ = 5 *Hz*, *f*_*a*_ = 70 *Hz*, SNR = 3 dB and coupling intensity = 0.7.

Examples from the two synthesized data sets shown in Fig. 1.

#### EEG data

The proposed PAC measure was evaluated on an EEG dataset from a previously published cognitive control-related error processing study^54^. A letter version of the speeded-reaction Flanker task^60^ was designed following the experimental protocol approved by the Institutional Review Board (IRB) of the Michigan State University. The data collection was performed in accordance with the guidelines and regulation established by this protocol. Written and informed consent was collected from each participant before data collection. In this paper, data from 19 participants were considered. A string of five letters, which could be congruent (e.g., SSSSS) or incongruent (e.g., SSTSS), was visually presented to each participant for each trial. The participants have to choose the center letter with a standard mouse with respect to the Flanker letters. The trials began with a 35 ms of flanking stimuli (e.g., SS SS). The target stimuli were then embedded in the center of the flankers (e.g., SSSSS/SSTSS) and remained for 100 ms (total presentation time is 135 ms) followed by an inter-trial interval of varying duration ranging from 1200 to 1700 ms. The trials were conducted to study the Error-Related Negativity (ERN) after an error response and the Correct-Related Negativity (CRN) after a correct response. Total number of trials was 480 in which the total number of error trials in different participants varied from 20 to 61. To keep the comparison between ERN/CRN consistent, the same number of correct responses was chosen randomly from the correct trials. Continuous EEG responses were recorded using the ActiveTwo system (BioSemi, Amsterdam, The Netherlands) by placing the 64 Ag–AgCl electrodes in accordance with the international 10/20 system. All EEG signals were sampled at 512 Hz. The trials with artifacts were removed, and the volume conduction was minimized using the Current Source Density (CSD) Toolbox^61^. The artifact removed data averaged over all trials were considered for the analysis in this paper.

### Time-frequency phase amplitude coupling using MVL (tf-MVL)

Let *x*(*t*) be the signal of interest and *f*_*a*_ be the high frequency, and *f*_*p*_ be the low frequency of interest. PAC is defined as the modulation of the high frequency amplitude, ${A}_{{f}_{a}}(t)$, with the phase, ${\varphi }_{{f}_{p}}(t)$, of the low frequency components. The first step is to extract the envelope of the high frequency amplitude signal and the phase of the low frequency signal. In this paper, we will use a recently introduced complex time frequency distribution, named Reduced Interference Rihaczek (RID-Rihaczek) time-frequency distribution^53^, to extract both the phase and amplitude components of *x*(*t*) within the frequency bands of interest.

Rihaczek defined the signal energy distribution in time and frequency using complex signal formulation. This definition results in a complex energy distribution based on the interaction energy at a frequency of interest, *f*, within some frequency band and at a given time, *t*, within an infinitesimal time interval as^62^:

4 $$C(t,f)=x(t){X}^{\ast }(f){e}^{-j2pft},$$

where *x*(*t*) is the signal and *X*(*f*) is its Fourier transform. The complex energy density function provides a better description of the properties of phase-modulated signals that are not available from conventional time-frequency distributions. While the time-frequency resolution of the wavelet transform is determined by the wavelet function, for the Rihaczek distribution, this is determined by the rate of change of the instantaneous frequency which provides better localization for phase-modulated signals^53^. The Rihaczek distribution is a bilinear, time and frequency shift covariant time-frequency distribution that satisfies the marginals, preserves the energy of the signal with strong time and frequency support properties^62^. Therefore, the Rihaczek distribution is a complex time-frequency distribution that provides both a time-varying energy spectrum and a phase spectrum with good time-frequency localization.

As the Rihaczek distribution is bilinear, it suffers from cross-terms for multicomponent signals. This problem can be addressed by introducing the RID-Rihaczek distribution. RID-Rihaczek distribution is a modified version of the Rihaczek distribution that uses the Choi-Williams kernel to filter out the cross-terms and is given by^52^:

5 $$C(t,f)=\iint \exp (-\frac{{(\theta \tau )}^{2}}{\sigma }\exp (j\frac{\theta \tau }{\sigma })A(\theta ,\tau ){e}^{-j(\theta t+2\pi f\tau )}d\tau d\theta ),$$

where $\exp (j\frac{\theta \tau }{\sigma })$ corresponds to the kernel function for the Rihaczek distribution^62^, $\exp (-\frac{{(\theta \tau )}^{2}}{\sigma })$ corresponds to the Choi-Williams kernel, *A*(*θ*, *τ*) is the ambiguity function of the given signal *x*(*t*) defined as:

6 $$A(\theta ,\tau )=\int x(u+\frac{\tau }{2}){x}^{\ast }(u-\frac{\tau }{2}){e}^{j\theta u}du.$$

This distribution still belongs to Cohen’s class of distributions and reflects both the time-varying energy and the phase of the signal^52^. The kernel functions can be thought of as two-dimensional lowpass filters that act on the ambiguity function that captures the time-varying autocorrelation of the signal. Through the kernel function, it is possible to reduce the effect of the cross-terms and localize the energy and phase estimates.

RID-Rihaczek distribution has been previously employed for estimating phase and computing phase synchrony within a frequency band^52,53^. It has been shown that RID-Rihaczek based phase synchrony estimates have a lower bias, better robustness to noise and higher time-frequency resolution compared to conventional methods including the Hilbert and wavelet transforms^52,53^. In the case of PAC, RID-Rihaczek can be used to extract both the envelope of the high frequency amplitude component, ${A}_{{f}_{a}}(t)$, and the low frequency phase component, ${\varphi }_{{f}_{p}}(t)$. In the following, we will provide analytical expressions for ${A}_{{f}_{a}}(t)$ and ${\varphi }_{{f}_{p}}(t)$ extracted from the Rihaczek distribution. These results can be easily extended to RID-Rihaczek distribution through a convolution with the kernel function.

To extract ${A}_{{f}_{a}}(t)$, we propose a frequency constrained time marginal as follows:

7 $${A}_{{f}_{a}}(t)={\int }_{{f}_{{a}_{1}}}^{{f}_{{a}_{2}}}C(t,f)df,$$

where ${f}_{{a}_{1}}$ and ${f}_{{a}_{2}}$ define the bandwidth around the high frequency of interest, *f*_*a*_. As RID-Rihaczek distribution preserves the total energy and satisfies the time marginal, i.e. ${\int }_{-\infty }^{\infty }\,C(t,f)df=|x(t){|}^{2}$, (7) yields an accurate estimate of the signal envelope in the given frequency band. For an analytic signal, *x*(*t*) = *A*(*t*)*e*^*jϕ*(*t*)^ with Fourier transform *X*(*f*) = *B*(*f*)*e*^*jθ*(*f*)^, the instantaneous amplitude estimate based on the Rihaczek distribution can be derived as^52^:

8 $$\begin{array}{rcl}{A}_{{f}_{a}}(t) & = & A(t){e}^{j\varphi (t)}{\int }_{{f}_{{a}_{1}}}^{{f}_{{a}_{2}}}\,B(f){e}^{-j\theta (f)}{e}^{-j2\pi ft}df,\\  & = & A(t){e}^{j\varphi (t)}[A(t){e}^{-j\varphi (t)}\ast sinc(({f}_{{a}_{2}}-{f}_{{a}_{1}})t]{e}^{j\frac{{f}_{{a}_{1}}+{f}_{{a}_{2}}}{2}t},\end{array}$$

where the second equality is obtained using the multiplication property of Fourier transform, i.e. multiplication in the frequency domain is equivalent to convolution in the time domain. As ${f}_{{a}_{2}}-{f}_{{a}_{1}}$ increases, the *sinc* function becomes a Dirac delta function, *δ*(*t*), and ${A}_{{f}_{a}}(t)=|A(t){|}^{2}$. Therefore, the amplitude estimate obtained from Rihaczek distribution is a smoothed version of |*A*(*t*)|^2^ around *f*_*a*_. In (8), *f*_*a*_ is any frequency and does not have to be known a priori. The amplitude estimate is automatically computed for all frequencies as part of the time-frequency distribution computation. Similar to GMW, the frequencies of interest will have higher amplitude estimates. It should be noted that, unlike Hilbert and wavelet based methods, the envelope estimate based on time-frequency distribution is a quadratic function of the signal.

If the high frequency amplitude of the signal is coupled to the phase of a low frequency oscillation at *f*_*p*_, then this time marginal ${A}_{{f}_{a}}(t)$ is expected to generate a peak at *f*_*p*_. This detected frequency is selected as the low frequency that is coupled with the amplitude of the high frequency component and the phase information at that frequency is extracted from the complex time-frequency distribution as follows:

9 $${\varphi }_{{f}_{p}}(t)={\rm{\arg }}[\frac{C(t,{f}_{p})}{|C(t,{f}_{p})|}].$$

For an analytical signal, *x*(*t*) = *A*(*t*)*e*^*jϕ*(*t*)^ with Fourier transform *X*(*f*) = *B*(*f*)*e*^*jθ*(*f*)^, the time-varying phase estimate based on the Rihaczek distribution can be derived as^52^:

10 $$\begin{array}{rcl}{\varphi }_{{f}_{p}}(t) & = & {\rm{\arg }}\,[\frac{A(t){e}^{j\varphi (t)}B({f}_{p}){e}^{-j\theta ({f}_{p})}{e}^{-j2\pi {f}_{p}t}}{A(t)B({f}_{p})}],\\  & = & {\rm{\arg }}\,[{e}^{j\varphi (t)}{e}^{-j\theta ({f}_{p})}{e}^{-j2\pi {f}_{p}t}],\\  & = & \varphi (t)-\theta ({f}_{p})-2\pi {f}_{p}t,\end{array}$$

where *ϕ*(*t*) and *θ*(*f*) refer to the phase in the time and the frequency domains, respectively.

After detecting the amplitude and phase, we can use any existing method such as mean vector length (MVL)^10^, modulation index (MI)^27^ or phase locking value (PLV)^51^. As Hülsemann *et al*. suggested, MVL is more suitable for high SNR data as it is more sensitive to coupling strength and width compared to other two methods^41^. Therefore, in this paper, the coupling between *f*_*p*_ and *f*_*a*_ is quantified by using MVL method. This approach estimates PAC from a signal of length *N*, by mapping phase time series ${\varphi }_{{f}_{p}}(t)$ given by (9) and amplitude time series ${A}_{{f}_{a}}(t)$ given by (8) to a complex-valued vector at each time point, *t*^10^. To quantify the coupling between *f*_*p*_ and *f*_*a*_, MVL method measures the length of the average vector and computes PAC^10^ as follows:

11 $$MVL({f}_{a},{f}_{p})=|\frac{1}{N}\mathop{\sum }\limits_{t=1}^{N}\,{A}_{{f}_{a}}(t){e}^{j{\varphi }_{{f}_{p}}(t)}|.$$

**Figure 2:**
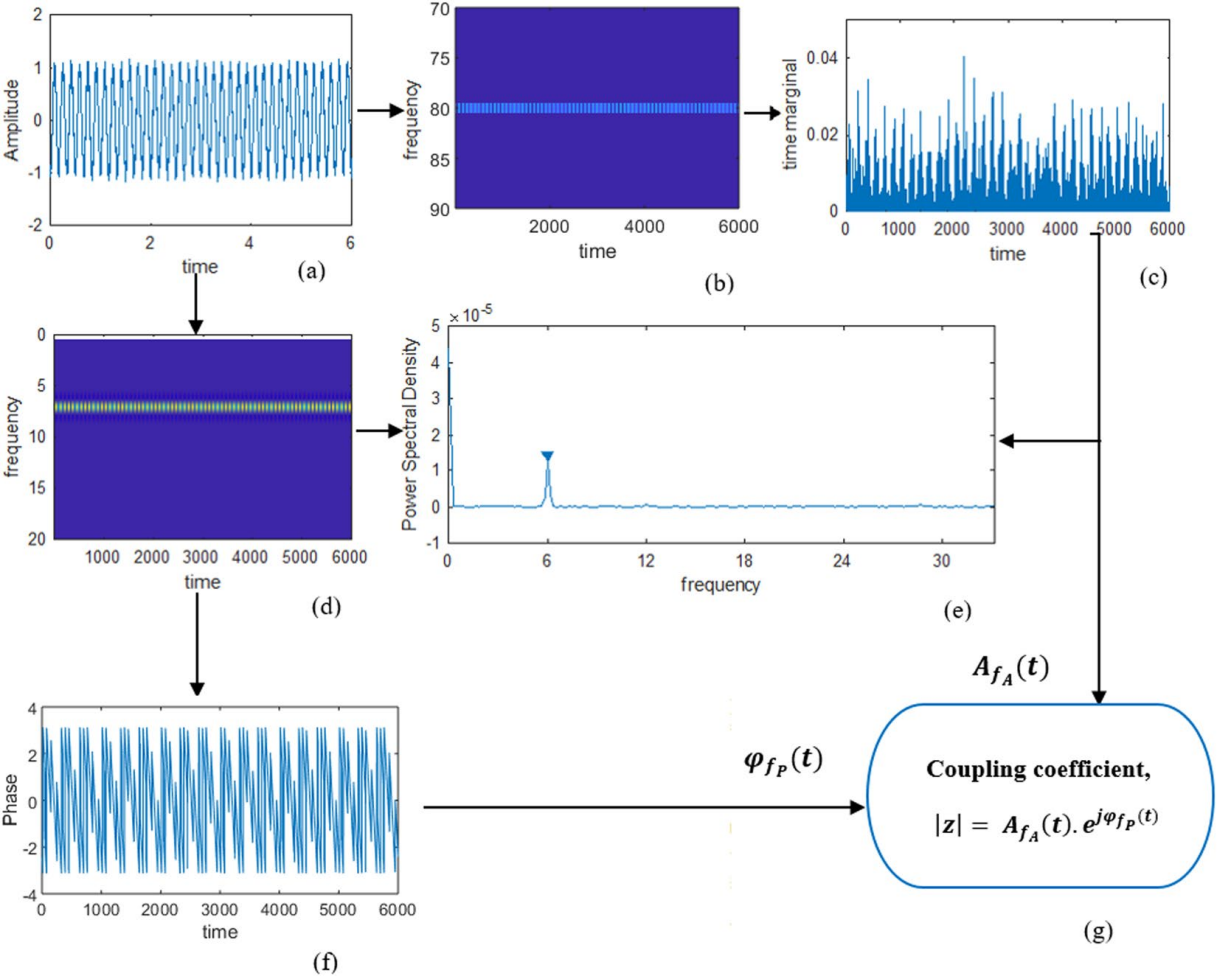
Illustration of the computation of phase-amplitude coupling on synthesized data. This synthesized data was generated by modulating the amplitude of an 80 Hz high frequency signal with the phase of a 6 Hz low frequency signal. (**a**) The synthesized signal; (**b**) Time-frequency component at high frequency; (**c**) Time marginal of the high frequency signal; (**d**) Time-frequency component at low frequency; (**e**) Illustration of detection of peak at coupled frequency from power spectral density of high frequency time marginal; (**f**) Phase component of the detected coupled low frequency component; (**g**) PAC measurement using the MVL metric.

A graphical representation of the proposed approach is given in Fig. 2. The Matlab scripts for implementing the tf-MVL are available online (https://github.com/muntam/TF-PAC).

### Significance testing

The statistical significance of the tf-MVL measure was determined by generating surrogate datasets. Following the guidelines in Aru *et al*.^42^, we generated surrogate datasets using a block swapping approach. This method ensures that phase distortion is minimized, reducing the false-positive rate. A permutted signal was generated by splitting the envelope of the high frequency oscillation, ${A}_{{f}_{a}}(t)$ at a random time point and then swapping the two resulting time series. MVL values are then computed by associating the permuted ${A}_{{f}_{a}}(t)$ with the original phase of the low frequency oscillation ${\varphi }_{{f}_{p}}(t)$. This procedure of block swapping the amplitude time series and matching it with the original phase time series was performed 200 times to obtain the surrogate MVL values. This approach is known to produce relatively conservative evaluations of statistical significance of PAC measures^42^. This procedure preserves the global statistical characteristics of the data while disordering the correspondence between phase and amplitude time series and generating a sequence of MVLs observed under the null hypothesis that phase-amplitude coupling is due to chance. The observed tf-MVL value was deemed to be significant only when it is larger than 95% of the surrogate tf-MVL values.

As the proposed time-frequency measure uses a quadratic distribution, the phase and amplitude estimates may be non-local causing a bias in the MVL estimates. However, these non-localities are minimized through the Choi-Williams kernel used in the definition of RID-Rihaczek distribution in (5). Moreover, Cohen *et al*.^44^ assert that the bias in MVL estimates due to non-stationarities in the signals can be mitigated by applying shuffling based permutation or debiasing terms^63,64^.

## Results

### Comparison of tf-MVL comodulogram with conventional Hilbert-MVL and Wavelet-MVL comodulogram

First, we compare the performance of three different approaches, i.e. Hilbert transform, wavelet transform and the proposed time-frequency approach, for computing PAC as reflected by the MVL comodulograms. The comparison of these three methods was performed in terms of both comodulograms and Shannon entropy. Comodulogram is a 2-D map that indicates the strength of coupling, as measured by (11), between different oscillation frequencies, with the *f*_*p*_ values along the x-axis, and *f*_*a*_ values along the y-axis^27^. As the coupling strength is plotted as a function of low and high frequency, comodulogram maps the coupling strength for (*f*_*a*_ and *f*_*p*_) and exhibits highest value for the combination with highest PAC. In all these cases, the comodulograms were constructed by considering a frequency step size of 1 *Hz* for *f*_*a*_ and 1 *Hz* for *f*_*p*_. For the comparison, a synthesized signal was generated following the synthesized dataset 1 method described above with high frequency *f*_*a*_ = 60 Hz, low frequency *f*_*p*_ = 10 Hz, coupling coefficient = 0.8, and SNR = 6 dB.

The proposed method is first compared with the conventional Hilbert transform based method^41,63,65,66^. The filter designed for the Hilbert transform based approach was a fourth order bandpass Butterworth filter. The bandwidth of the bandpass filter is selected to be variable as discussed in^42^. The filters that extract the envelope of the high frequency oscillation, *f*_*a*_, should be wide enough to accomodate the center frequency ± the modulating *f*_*p*_. If this condition is not met, The PAC cannot be identified without fulfilling this condition even if present^45^. As a result, a variable bandwidth defined as ±0.4 times the amplitude frequency (e.g. for *f*_*a*_ = 40 *Hz*, the chosen bandwidth was 40 ± 0.4 × *f*_*a*_ = [24, 56]) was chosen, which has been shown to improve the detectability of PAC in literature for Hilbert transform^48,66^. The bandwidth for *f*_*p*_ was kept narrow (*f*_*p*_ ± 1 *Hz*) to extract the sinusoidal waveforms^66^. After filtering, Hilbert transform was performed to extract the instantaneous phase and amplitude components and finally PAC was calculated by computing the MVL for the Hilbert method (Hilbert-MVL).

Generalised Morse wavelet (GMW) based PAC^49^ was also computed for comparisons. This method generates complex valued time series through analytic wavelet transform from where phase and amplitude components can be extracted using this complex valued signal. As described in^49^, the PAC measure using generalised Morse wavelet largely depends on the value of the selected parameters such as *β* and *γ*. In this paper, we set the values of these parameters as *β* = 6 and *γ* = 3 as suggested in Nakhnikian *et al*.^49^. After extracting phase and amplitude components, we compute the MVL for generalized Morse wavelet (Wavelet-MVL) similar to tf-MVL and Hilbert-MVL.

**Figure 3:**
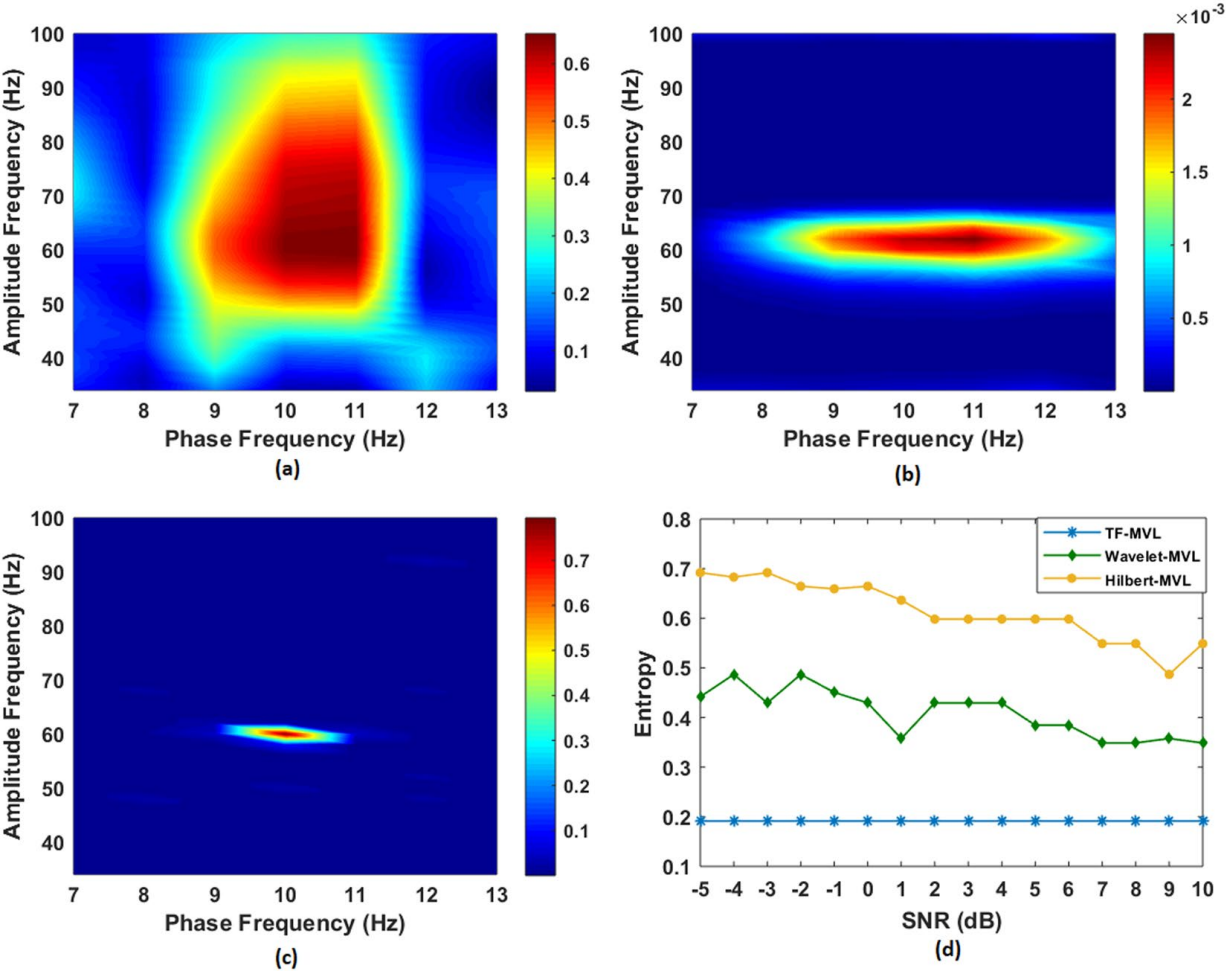
Comparison of proposed tf-MVL method with conventional PAC measures through comodulograms and Shannon entropy measure: (**a**) Hilbert transform based PAC (Hilbert-MVL) measure; (**b**) Generalized Morse Transform based PAC (Wavelet-MVL) measure and (**c**) Proposed time-frequency based PAC (tf-MVL) measure; (**d**) Comparison of Entropy Measure for Hilbert-MVL, Wavelet-MVL and tf-MVL comodulograms for varying SNR levels. A low entropy value corresponds to a comodulogram that is well-localized, while a high entropy value corresponds to a comodulogram that is distributed across a wide range of frequencies. The entropy for the tf-MVL method stays constant across different SNRs indicating the robustness of the method to noise.

The resulting comodulograms are illustrated in Fig. 3(a–c). As it can be seen from this figure, the tf-MVL method provides higher resolution estimates of the two frequencies that are coupled with each other compared to Hilbert transform and generalized Morse wavelet transform based methods. It can also be seen that GMW based method has better localization of the frequencies compared to the Hilbert transform. After computing the comodulograms, the three methods were also compared quantitatively using Shannon entropy to quantify the concentration of the comodulograms. The Shannon entropy for a comodulogram is defined in the same way that entropy is quantified for time-frequency distributions^67^, i.e. $H=-\sum _{{f}_{1},{f}_{2}}\,{p}_{{f}_{1},{f}_{2}}{\mathrm{log}}_{2}{p}_{{f}_{1},{f}_{2}}$, where ${p}_{{f}_{1},{f}_{2}}=\frac{MVL({f}_{1},{f}_{2})}{{\sum }_{{f}_{1}}\,{\sum }_{{f}_{2}}\,MVL({f}_{1},{f}_{2})}$. The comodulogram surface is first normalized to obtain a two-dimensional distribution similar to a probability density function and then the definition of Shannon entropy is applied to this surface. In information theory, a maximum value of entropy is obtained for a uniform distribution, whereas the minimum value is achieved for a Dirac delta function, i.e. when there is no uncertainty about the value of the random variable. In a similar fashion, for a comodulogram a high entropy value corresponds to a surface that is equally distributed across different frequency values while a low entropy value corresponds to a surface that is well-localized. Therefore, in this paper, entropy is used as a quantitative measure of concentration of the comodulogram surface similar to the way it has been applied to time-frequency distributions in prior work^67,68^. For different SNR values, the Shannon entropy of the comodulograms is given in Fig. 3(d). The lower Shannon entropy value for tf-MVL indicates that the MVL modulation index is more concentrated, i.e., has a higher resolution, and thus leads to more accurate PAC detection. Moreover, the entropy for the tf-MVL method stays constant across different SNRs indicating the robustness of the method to noise. In comparison, wavelet and Hilbert transform based methods have higher entropy, with Hilbert transform yielding the highest entropy, i.e. the worst localization. Moreover, both the wavelet and Hilbert transform based methods show a fluctuation of the entropy values across SNRs, indicating less robustness compared to time-frequency based method.

As the wavelet based method is fairly new and depends largely on the selection of the wavelet design parameters and the range of input frequencies, further research is required for the correct quantification of PAC using this approach. On the other hand, Hilbert transform based method is well-established. For these reasons, in the following sections, the proposed tf-MVL measure is compared to the conventional Hilbert-MVL approach in terms of the resolution of the comodulogram, accuracy of the estimated PAC value and robustness to varying signal parameters for both simulated and real data.

### Comparison of phase amplitude coupling indices (tf-MVL, tf-PLV and tf-MI)

**Figure 4:**
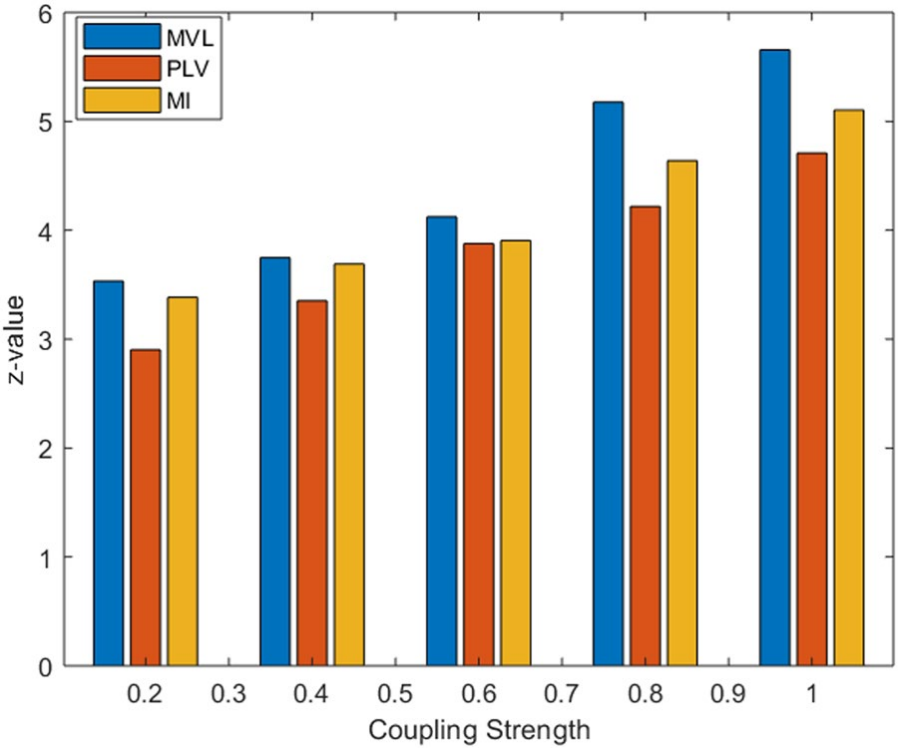
Performance comparison of the Phase Amplitude Coupling Measures tf-MVL, tf-PLV and tf-MI as a function of varying coupling strength. PAC values of all methods increased with increasing coupling strength and MVL differentiates best between different levels of coupling strengths.

To quantify the PAC, our proposed time-frequency based method can be used with any of the existing PAC indices such as MVL, PLV or MI, where the amplitude and phase are extracted using the proposed time-frequency method. To compare the performance of different PAC measures, tf-MVL, tf-PLV and tf-MI were computed as a function of varying coupling strength. The model described in synthesized dataset 1 was used for this comparison. Data was generated with *f*_*p*_ = 5 Hz, *f*_*a*_ = 70 Hz, SNR = 6 dB with a sampling frequency of 1000 Hz. Five different coupling strengths ranging from 0.1 to 1 with a stepsize of 0.2 were considered. In all of these cases, RID-Rihaczek has been used to extract the amplitude and the phase of the high and low frequency components, respectively, but different metrics have been employed to quantify the PAC. As shown in Fig. 4, the PAC values of all methods increase with increasing coupling strength.

In accordance with existing literature^41^, MVL is found to be more sensitive to the varying coupling strength, followed by MI and PLV. Despite small quantitative differences in these three measures, the same trend is observed for all three methods in the simulation. For this reason, all of the following analysis was performed using tf-MVL method.

### Comparison of accuracy of tf-MVL with Hilbert-MVL

Synthesized dataset 1 was used for this comparison as the ground truth for coupling strength is known. The data was generated with *f*_*p*_ = 5 Hz and *f*_*a*_ = 70 Hz with a sampling frequency of 1000 Hz. The range of interest for the high frequency was set to [40, 90] Hz whereas the low frequency range was chosen to be [2, 12] Hz. The two methods were compared by calculating the relative error rate of the estimated coupling strength using the following equation:

12 $$Relative\,Error(RE)=|\frac{PA{C}_{detected}-PA{C}_{actual}}{PA{C}_{actual}}|\times 100 \% .$$

**Figure 5:**
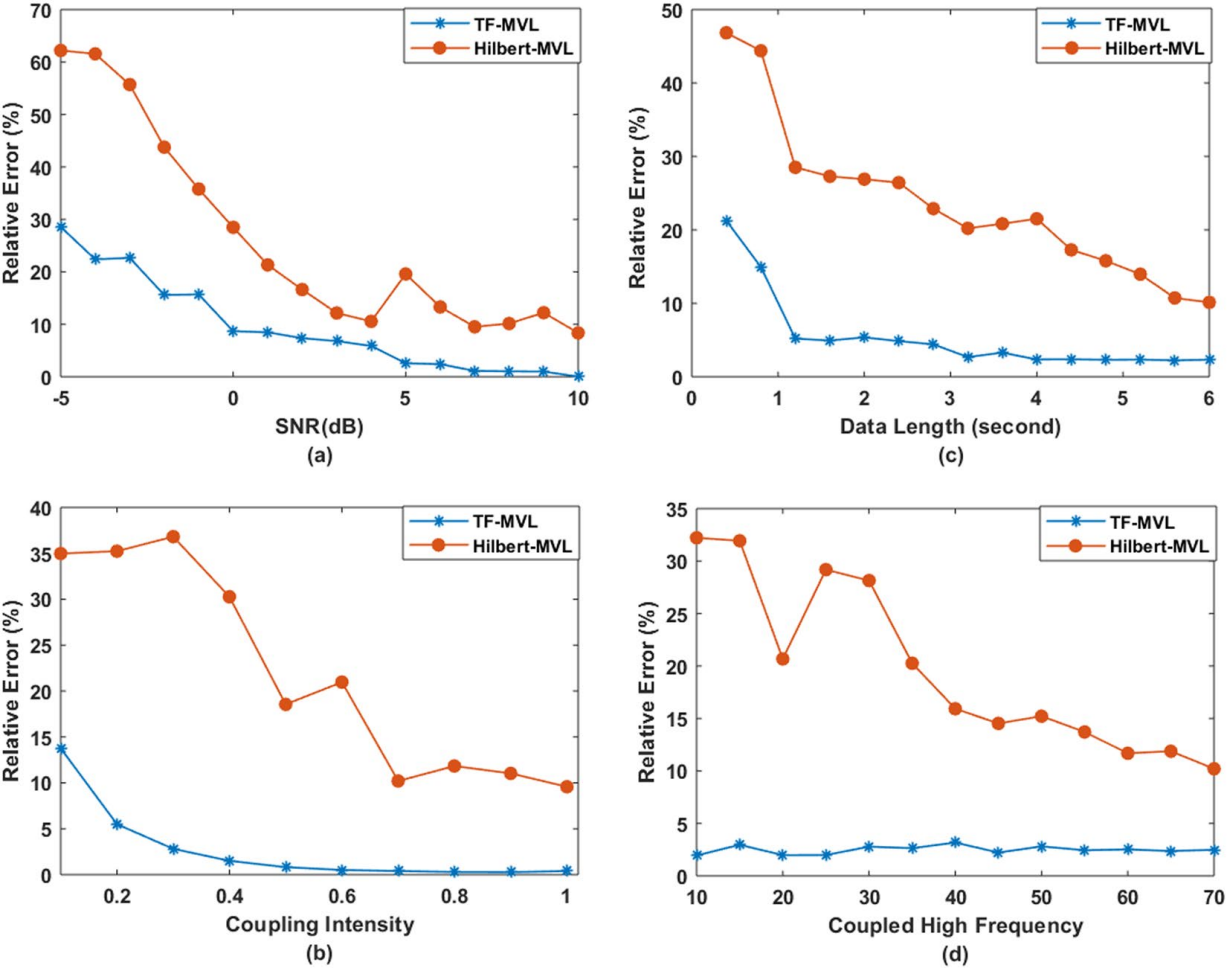
Performance comparison of tf-MVL measure with Hilbert Transform method for synthesized dataset 1; (**a**) Relative error for various SNR levels; (**b**) Relative error for various coupling strengths; (**c**) Relative error for different data lengths; (**d**) Relative error for coupled high frequency.

Figure 5 shows the comparison of the proposed method with Hilbert transform in terms of quantification of coupling coefficient under four different conditions: varying SNR from −5 to 10 dB, varying coupling strength of 0.1 to 1, varying data length of 0.4 to 6 seconds and varying the high frequency of 10 Hz to 70 Hz.

For varying SNR levels, it can be seen in Fig. 5(a) that the proposed method is more robust against noise with smaller errors in estimating the coupling parameter (Mean Error < 10%). Even at high noise levels, e.g., SNR = −5 dB, the error rate is as low as 28% for tf-MVL compared to 63% relative error exhibited by Hilbert transform based method.

For varying degrees of coupling strength, ten different coupling strengths ranging from 0.1 to 1 were considered. The relative errors in coupling strength estimation are depicted in Fig. 5(b). The proposed method is better at quantifying the true coupling strength compared to Hilbert transform based PAC. As tf-MVL does not depend on bandpass filtering, the performance of tf-MVL (Mean Error: 2.62 ± 3.27%) is much better at detecting the coupling strength compared to Hilbert Transform (Mean Error: 21.94 ± 11.36%).

The sensitivity of PAC to data length is evaluated in Fig. 5(c). Different signal lengths from 0.4 to 6 seconds were considered. For both methods, the accuracy of detecting the coupling strength improved with increasing signal length, although at different rates. Overall, for signal durations longer than one second, tf-MVL produced the best coupling strength estimates.

Finally, the two methods were compared with respect to the difference between the two frequencies by changing the high frequency amplitude providing signal. The *f*_*a*_ value was varied from 10 Hz to 70 Hz with an increment of 5 Hz by keeping the *f*_*p*_ value constant at 5 Hz. As shown in Fig. 5(d), the change of frequency has no effect on the proposed t-f based PAC measure resulting in consistently low error rates, whereas Hilbert transform performed better as the difference between the two frequencies got larger.

### Comparison of the robustness of tf-MVL with Hilbert-MVL

tf-MVL and Hilbert-MVL were compared for synthesized dataset 2 for different signal parameters including varying SNR levels, coupling strength, data length, high frequency component and narrowband vs. broadband coupling. In this case, as there is no ground truth for the coupling strength, instead of the relative error, the two methods were compared using the MVL values. Moreover, the PAC values computed from the surrogate data for both the tf-MVL and Hilbert-MVL methods were compared with the respective observed MVL values to determine the significance of the results.

**Figure 6:**
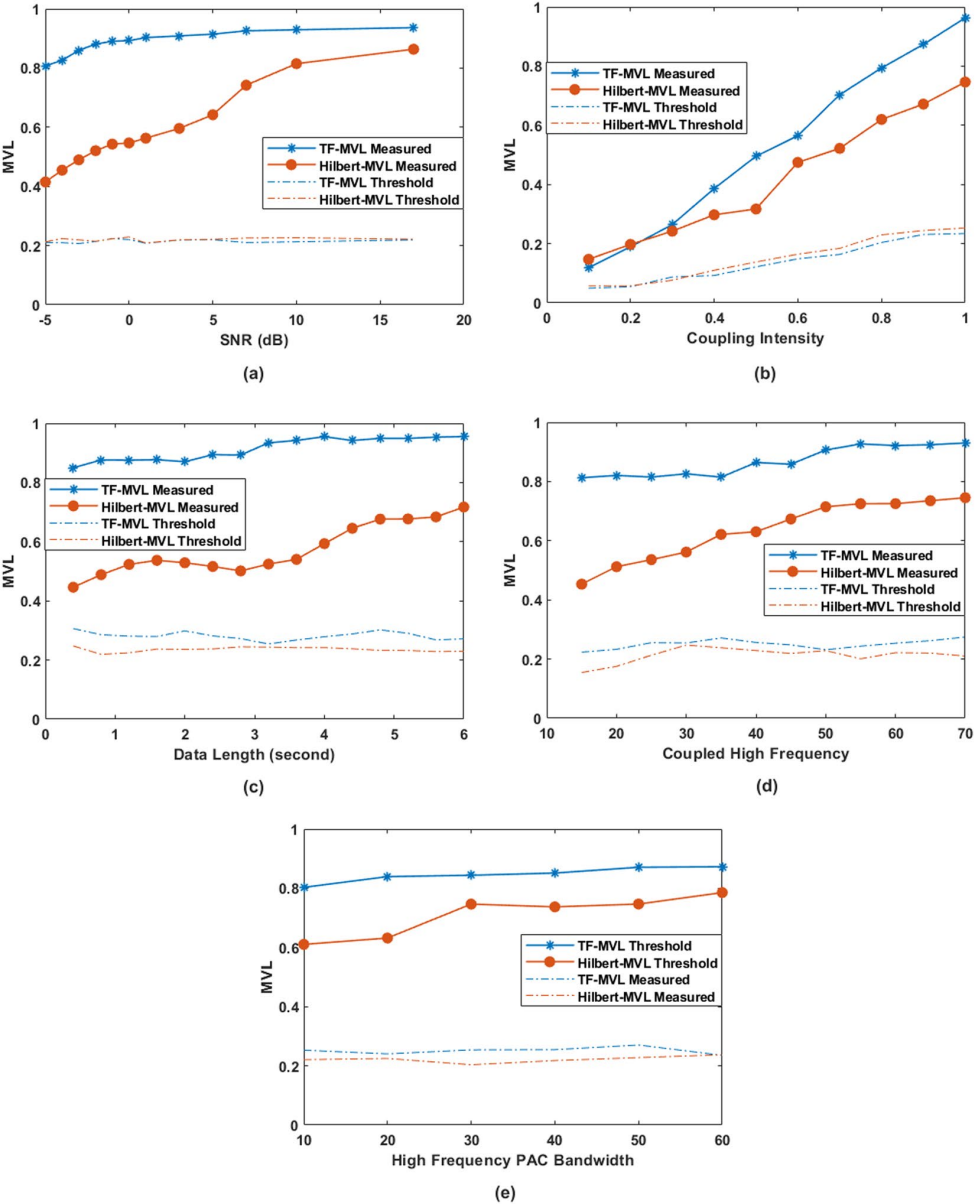
The robustness comparison of proposed tf-MVL measure with Hilbert-MVL method for synthesized dataset 2. The broken lines indicate the critical threshold for PAC (MVL value at relative cut off 5%) among 200 surrogate data. (**a**) MVL for various SNR levels; (**b**) MVL for various coupling strengths; (**c**) MVL for various data lengths; (**d**) MVL for different coupled high frequency components; (**e**) MVL for various bandwidths of coupled high frequency. Synthesized signals were generated with the low-frequency phase component at 5 Hz and a varying frequency range for the high-frequency component as [65–75], [60–80], [55–85], [50–90], [45–95] and [40–100] to create PAC with 10 Hz to 60 Hz amplitude bandwidth.

As shown in Fig. 6(a), the tf-MVL method was more robust than the Hilbert-MVL for different noise levels. The tf-MVL method reached the actual PAC value at 1 dB whereas the Hilbert-MVL method showed a high MVL value only at high SNR levels. Both tf-MVL and Hilbert-mvl showed an increase in MVL with an increase in coupling intensity. As shown in Fig. 6(b), tf-MVL method was more sensitive to the coupling strength compared to Hilbert-MVL. The effect of data length is shown in Fig. 6(c). The tf-MVL is more robust and reaches the level of acceptable PAC at 1.2 seconds compared to Hilbert-MVL which provides acceptable PAC only at 3.2 seconds, thus indicating that tf-MVL method is still effective when data length is very small. As shown in Fig. 6(d), the high frequency that the low frequency is coupled to has no effect on the tf-MVL method. As no filtering is required for tf-MVL, this method can detect PAC even when the frequency difference between high and low frequency is as low as 5 Hz. On the other hand, due to the required filtering in the implementation of the Hilbert-MVL method, this method can only start to detect the true coupling when the high frequency is at 40 Hz. In Fig. 6(e), we evaluated the effect of narrowband vs. broadband coupling. For this comparison, the *f*_*p*_ frequency was fixed at 5 Hz whereas the *f*_*a*_ frequency range was varied as [65–75], [60–80], [55–85], [50–90], [45–95] and [40–100] to generate coupling bandwidth ranging from 10 Hz to 60 Hz. The SNR was fixed at 6 dB with coupling strength fixed at 0.9. The variation of tf-MVL is low compared to Hilbert-MVL with varying coupling bandwidth. As expected, an increase in Hilbert-MVL was found with an increase in coupling bandwidth.

The threshold values obtained through surrogate data testing (MVL threshold for 95% confidence interval) are also shown in Fig. 6. As reflected in Fig. 6(a–e), for both tf-MVL and Hilbert-MVL cases the observed MVL was significantly higher (one tail Wilcoxon Signed Rank Sum Test, *p* < 0.05) than the threshold, thus rejecting the null hypothesis.

### tf-MVL for EEG data

**Figure 7:**
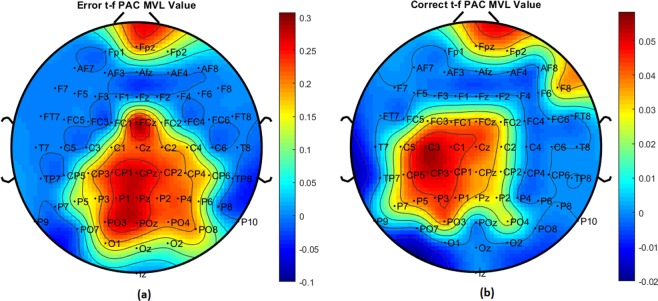
Topo plots of tf-MVL values for (**a**) ERN and (**b**) CRN response types.

The time-frequency based PAC measure was applied to EEG data collected during a cognitive control study where ERN and CRN were compared after error and correct responses, respectively. As previous studies indicate increased synchronization associated with the ERN in the time window 25–75 ms^53,54^, all analysis was performed for the 25–75 ms time window of the EEG data. For each electrode, PAC was computed between a band of low frequency oscillations (*f*_*p*_) in the [2, 13] Hz range, and high frequency oscillations (*f*_*a*_) in the [34, 100] Hz range. The average coupling strength was calculated for 66 frequencies distributed linearly in the *f*_*a*_ range of interest with an increment of 1 Hz. The topoplots showing the computed tf-MVL value for all 64 electrodes for both ERN and CRN are given in Fig. 7. From this figure, it can be seen that the PAC averaged across subjects is higher for ERN compared to CRN and that the high PAC values for ERN are concentrated around FCz and central-parietal regions.

**Figure 8:**
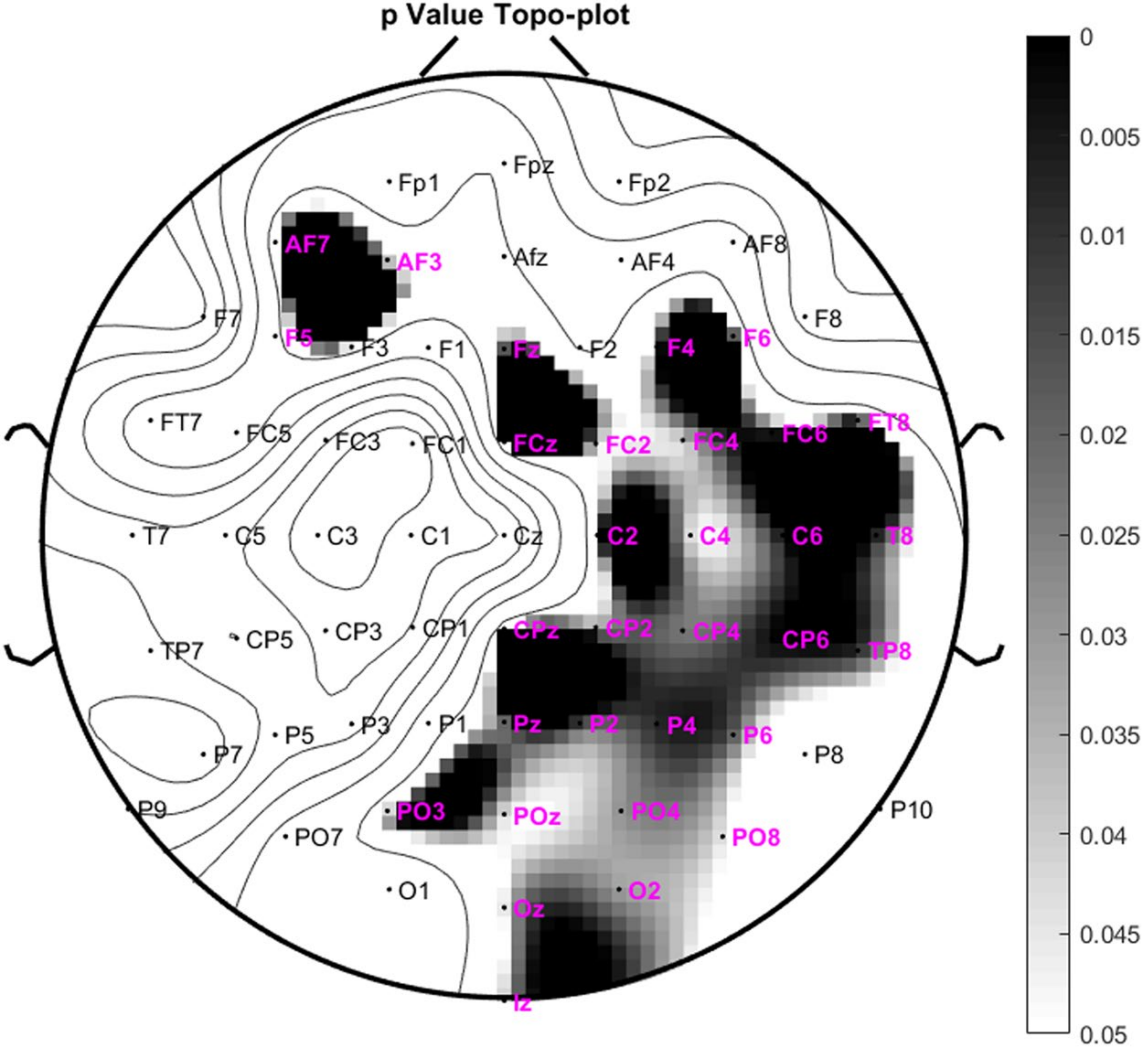
p-value topo-plots for ERN and CRN after error and correct response types respectively. The channels marked in pink showed significant difference between ERN and CRN (Wilcoxon Signed Rank Sum Test with p < 0.05).

A Wilcoxon Signed Rank Sum Test is conducted to determine the statistical significance of the difference between ERN and CRN PAC values across all electrodes. tf-MVL value is significantly higher (*p* = 0.00381) for ERN compared to CRN. A *p*-value topoplot that shows the channels with significant difference between ERN and CRN is given in Fig. 8. The most significant difference was found for FCz with *p* = 0.000367 after Bonferroni correction.

**Figure 9:**
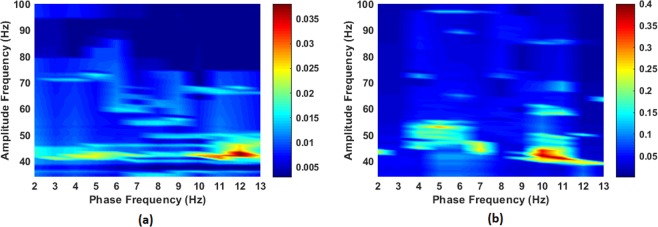
Comodulograms show the PAC between low and high frequency bands for (**a**) CRN and (**b**) ERN of EEG data for FCz channel.

As FCz provided the highest tf-MVL value and the highest significant difference between the two response types, the coupling strength at FCz is depicted as a comodulogram in terms of *f*_*a*_ vs. *f*_*p*_, for both error and correct responses in Fig. 9. The overall strongest PAC index was found between the phase of alpha (9–12 Hz) and the amplitude of slow gamma (40–60 Hz) oscillations (Fig. 9). Though both ERN and CRN responses exhibited high PAC for these ranges of frequencies, the coupling coefficient for ERN (*MVL* = 0.22424 ± 0.1039) was significantly greater compared to the coupling coefficient for CRN (*MVL* = 0.0.06758 ± 0.03579) with a *p* = 0.0031 determined by a Wilcoxon Signed Rank Sum Test.

## Discussion

Human brain is known to route information through multiple networks operating in parallel. Oscillatory coupling across frequency bands provides the temporal and spatial dynamics necessary to enable these networks. One prominent type of cross-frequency coupling known as phase-amplitude coupling may reflect integration processes across populations of neurons. PAC has been reported to control the long-distance communication considering that the slow oscillations of the neuronal signal can propagate at larger scales compared to the fast ones^9,11,14^ and thus was proposed as “canonical mechanism for neural syntax”^40^. Although there is a growing literature on measures to quantify PAC from neurological data, the best approach to detect and quantify the phenomena is still difficult to settle on.

In this paper, we proposed a time-frequency based PAC as a novel method for estimating cross-frequency phase-amplitude coupling and illustrate that the proposed method offers higher accuracy and robustness compared to existing methods through simulations. We first tested the model on two different sets of simulated signals, allowing us to generate a fully controlled comparison of models and parameters and then applied the proposed tf-MVL model to estimate the PAC from neurophysiological signals. The results from synthesized data showed that the proposed time-frequency based PAC method provides several advantages over existing Hilbert and Wavelet-MVL methods. First, the proposed method estimates both the amplitude of the high frequency signal component and the phase of the low frequency component directly from a high resolution complex time-frequency distribution. Using properties of Cohen’s class of distributions, the proposed method can extract high resolution estimates of both the envelope and the phase of the oscillations resulting in high resolution comodulograms. Unlike Hilbert transform based methods, the proposed method does not require bandpass filtering of the signals, and thus overcomes the problems of misidentification of the frequencies due to the selection of the bandwidth and the transition band of the bandpass filter. While analytic wavelet transform based PAC methods address some of the shortcomings of Hilbert transform based PAC estimates, they are highly dependent on the selected wavelet parameters and the user provided range of frequencies. Unlike wavelet based PAC measures, the proposed approach does not depend on any design parameters and offers uniform frequency resolution thus the estimated PAC comodulograms do not change with the range of frequencies considered. Second, the proposed PAC measure is more robust to varying signal conditions such as noise level, signal length, coupling strength and the separation between the coupled frequencies. In all of these cases, tf-MVL is more accurate at estimating the true coupling value. Finally, the proposed measure has been shown to be effective in detecting significantly higher alpha-gamma PAC for ERN compared to CRN. This finding is consistent with the results from previous studies^66,69,70^, where an error related MEG study showed significant alpha gamma coupling. Dynamic coupling between alpha phase (8–13 Hz) and slower gamma amplitude (<40 Hz) has also been reported during visual processing in prior research^63,70,71^. It is hypothesized that during a visual task, the ongoing gamma-band activity in the visual cortex becomes temporally segmented by different phases of alpha-band activity^63,70^. Moreover, the coupling strength was found to be higher for ERN compared to CRN, which is in line with the finding reported by Cohen *et al*.^72^. This can be explained by the fact that large-scale functional integration across different frequency bands supports flexible behavior adaption to improve the performance after an error^73^. The difference between ERN and CRN was most prominent at FCz, which is centered around the medial frontal cortex. This region was reported to be active during error processing and negative feedback^72^ as it is hypothesized that error initiates the medial frontal based top-down control mechanisms to improve the performance^74,75^. Botvinick *et al*. also reported that medial frontal cortex could detect error and conflicts and send signals to the lateral prefrontal cortex that behavioral adaptation is needed^76^. Thus, our findings are consistent with previous literature implicating higher alpha-gamma PAC in the medial frontal cortex and relating this with error-related performance.

Some of the limitations of the proposed time-frequency based amplitude and phase estimation are as follows. First, the proposed method is based on a nonlinear quadratic transform of the signal as opposed to the Hilbert or wavelet transform based methods which are linear. As such, the computational complexity of the proposed method is ${\mathscr{O}}({N}^{2}\,\mathrm{log}\,N)$ compared to Hilbert transform which has a complexity of ${\mathscr{O}}(N\,\mathrm{log}\,N)$. Recently, it has been shown that quadratic time-frequency distributions can be approximated using linear transforms such as the short-time Fourier transform, thus obtaining computational complexity close to ${\mathscr{O}}(N\,\mathrm{log}\,N)$^77^. It is also worth mentioning that this computational complexity comes with the benefit that the proposed method does not depend on the choice of any parameters. The only parameter that can be tuned in the computation of RID-Rihaczek distribution is *σ* which controls the trade-off between resolution and cross-terms. In this paper, *σ* = 0.001 was selected for all simulated and real data examples as it was observed that *σ* did not have a significant effect on the PAC values. In comparison, the Hilbert transform requires the choice of the filter parameters and the wavelet transform requires the choice of the parameters, *β* and *γ*, which greatly affect the amplitude and phase estimates. Second, the proposed method is more memory intensive compared to linear methods as a complete time-frequency surface needs to be stored for extracting the amplitude and phase components at frequencies of interest. Due to this increased memory requirement and high computationally complexity, the proposed method can be applied to long epochs of neurophysiological recordings through a sliding window approach. Third, the proposed method similar to existing PAC metrics is sensitive to data length. Simulation results indicate that the accuracy of the PAC value improves after 1000 samples. This is very similar to the behavior of the Hilbert transform based method. As such, the proposed method does not require more data samples than Hilbert transform to obtain accurate PAC estimates. Finally, the method presented in this paper focuses on PAC within a signal, thus computing univariate PAC. However, most neurophysiological recordings involve data from multiple channels. Moreover, as indicated by de Cheveigne *et al*., a recording at a single electrode does not necessarily correspond to a single brain dynamic^78^. Therefore, a multivariate analysis is necessary to determine the cross-frequency interactions across channels as well as to separate the PAC within a channel from the nearby neural activity. Cohen^44^ suggested a generalized eigenvalue decomposition based approach to address the latter. However, a complete bivariate PAC functional connectivity network analysis similar to bivariate PLV based connectivity networks^54^ is still missing. The proposed univariate tf-MVL method can be easily extended to the bivariate case by computing PAC between all possible electrode and frequency pairs. This would result in a complete representation of the multi-frequency functional connectivity networks of the brain^79,80^. The proposed tf-MVL measure can also be extended to consider different modes of coupling such as a biphasic coupling.

## Data Availability

The datasets generated during and/or analysed during the current study are available from the corresponding author on reasonable request.
